# Household Water and Handwashing Facilities and Early Childhood Education Participation in Nepal: Evidence From a Nationally Representative Cross‐Sectional Survey

**DOI:** 10.1002/hsr2.72254

**Published:** 2026-04-02

**Authors:** Md Salek Miah, Fhameda Faija Lamia, Mst. Labonna Begum, Md Shied, Rima Akhter

**Affiliations:** ^1^ Department of Statistics Shahjalal University of Science and Technology Bangladesh; ^2^ Department of Computer Science and Engineering Sylhet Engineering College Bangladesh; ^3^ Computer Science Brunel University London UK; ^4^ Shahabuddin Medical College Bangladesh; ^5^ School of Public Health University of Saskatchewan Saskatoon Canada

**Keywords:** ECE, LMICs, spatial, stratified, WASH

## Abstract

**Background and Aims:**

Early childhood education (ECE) is crucial for cognitive, social, and emotional development. Household water, sanitation, and hygiene (WASH) may influence ECE enrollment, but their role in Nepal is unclear. This study examined associations between household water and handwashing facilities and ECE participation among children aged 3–4 years, accounting for rural–urban and socioeconomic factors.

**Methods:**

This cross‐sectional study used nationally representative secondary data from the Nepal Demographic and Health Survey (NDHS) 2022, including 1057 children aged 3–4 years. Descriptive analyses, survey‐weighted multivariable logistic regression, and stratified analyses were employed according to rural‐urban residence. Additionally, spatial mapping was used to examine geographical variation in ECE participation. Model performance was evaluated using receiver operating characteristic (ROC) curve analysis and the area under the curve (AUC). All results were reported as adjusted odds ratios (AORs) with 95% confidence intervals (CIs).

**Results:**

Overall, 78.7% of children participated in ECE. Improved water sources, treated water, and handwashing facilities were associated with higher enrollment in bivariate analyses. After adjustment, household media access (aOR:5.17; 95% CI:2.04–13.10) and urban wealth (aOR: 2.61; 95%CI:1.04–6.52) remained significant. The predictive model showed excellent discrimination (AUC = 0.957). Provincial disparities existed, with Karnali and Koshi higher and Gandaki lower.

**Conclusion:**

Media contact drives ECE enrollment, whereas WASH effects appear attenuated after accounting for socioeconomic and maternal factors. Improving maternal education and media‐based awareness may enhance ECE participation, especially in rural areas.

## 1 Introduction

Early childhood development (ECD) is defined as the cognitive, social, emotional, and motor development of the child in the first years of life [1]. It is an important period for brain development and builds the foundation for lifelong learning, health, and well‐being [2]. Early childhood education (ECE) is defined as the organized group activities of trained personnel with an organized curriculum to promote comprehensive development and to provide preparation for formal schooling [3]. The United Nations Educational, Scientific, and Cultural Organization (UNESCO) describes ECE as dealing with the social, emotional, cognitive, and physical demands to provide a foundation for continuous learning and health for life [4].

Nevertheless, despite the important role of ECE, there is a low rate of worldwide enrollment in ECE, with only 40% of kids involved in organized ECE services before the age of entry into the formal education system of the primary school age group [5]. It has been noted that enrollment levels are particularly low in South Asia, including countries such as Nepal and Bangladesh, with considerable variation across and within countries [6, 7]. In Nepal, the government has expanded early childhood education through community‐based ECD centers and trained facilitators to improve school readiness and child development outcomes [8]. Over the past decade, the Government of Nepal has increasingly integrated early childhood education within the national education framework through the expansion of Early Childhood Education and Development (ECED) centers and trained facilitators [9]. These initiatives have substantially increased enrollment and access to early learning services across the country [10]. Despite these policy efforts, recent evidence indicates that disparities in participation persist across geographic regions and socioeconomic groups, highlighting the need to better understand the determinants of ECE enrollment [11, 12].

Water, sanitation, and hygiene (WASH) accessibility is an integral element affecting the welfare and change in children's health status [13]. According to the WHO/UNICEF Joint Monitoring Programme (JMP), “Basic household WASH” is defined as having all of the following: (i) an improved water source accessible within a 30‐min round trip journey, (ii) an improved and unshared sanitation facility, and (iii) a handwashing station with soap and water. “Limited household WASH” refers to households that meet some but not all of these criteria, while “Improved household WASH” refers to households that have access to safely managed water, improved sanitation, and handwashing facilities according to JMP definitions [14]. Improved WASH effectively prevents infections and absenteeism and indirectly protects and supports those affected or taking part in educational activities [15]. Globally, about 82% of the population has access to improved sources of water. However, sanitation coverage is lower, with large disparities in regions and within socio‐economic categories [16]. In the South Asia region, the situation is worse for countries like Bangladesh and the massive population base in India [17]. Improvements in Nepal are patchy across the administrative divisions of the province and by economic quintiles and rural‐urban differences, affecting possible discrepancies in child development [18].

However, various studies among low‐ and middle‐income countries (LMICs) have established the important predictors of ECE attendance [19]. These are child characteristics (age and health status), mother's characteristics (education and employability), household wealth, and urban status [1, 20]. In Bangladesh, household water, sanitation, and hygiene, among others, are established predictors of ECE attendance, along with mothers' education and household wealth [7]. Childhood diseases, malnutrition, and poor hygiene practices also lower attendance and contribute to learning inequities [21].

Interventions aimed at improving WASH conditions in educational environments in LMICs have shown benefits in reducing hygiene‐related diseases and improving school attendance [22, 23]. However, comparatively less attention has been given to the role of household‐level WASH conditions in shaping participation in early childhood education, particularly at subnational levels [24]. Evidence from Nepal remains limited, as most studies focus on national aggregates or school‐aged children rather than early childhood education at the national level [6, 25]. Furthermore, few studies have explored provincial‐level disparities or spatial patterns in ECE participation, despite Nepal's diverse geographical and socioeconomic landscape [26, 27].

In early childhood, safe household water and functioning handwashing facilities are especially crucial for preventing infections and supporting health development [28]. Adequate access to safe handwashing with soap can reduce illness‐related absenteeism, improve child health, and enhance readiness for participation in early childhood education programs [29]. Thus, the household water environment and hand hygiene conditions may comprise key yet underexplored determinants of ECE participation in LMICs settings such as Nepal [30].

In this study, a conceptual framework is adopted where household WASH conditions may influence ECE participation through several pathways. Improving water and hygiene conditions may reduce childhood illness and enhance learning readiness, while maternal education, household wealth, and residence may shape access to both WASH resources and educational opportunities [25]. Given the cross‐sectional nature of the data, the study focuses on statistical associations rather than causal relationships. It is hypothesized that children from households with better access to water and hygiene facilities are more likely to participate in ECE programs, even after accounting for maternal and socioeconomic characteristics [31]. In addition, the study explores rural–urban and provincial disparities to better understand geographical inequalities in ECE participation across Nepal [18, 32].

Therefore, the objective of this study is to assess the association between household water and handwashing facilities and early childhood education participation among children aged 3–4 years in Nepal, while accounting for socioeconomic, maternal, and geographical factors, including rural–urban and provincial differences.

The study aligns with global development priorities, including United Nations Sustainable Development Goal (SDG) 4.2, which aims to ensure universal access to quality early childhood development and education, and SDG 6, which focuses on universal access to safe water, sanitation, and hygiene services. The findings are expected to provide evidence to support policymakers and development partners in designing targeted interventions such as improved household WASH access, awareness programs, and geographically targeted education policies to enhance ECE participation and reduce disparities in Nepal [33].

## 2 Methodology

### 2.1 Study Design and Data Source

This study used a cross‐sectional design and secondary data from the nationally representative Nepal Demographic and Health Survey (NDHS) 2022, which includes household records, maternal, child, and all demographic records, etc. The NDHS is a nationally representative survey implemented under the global Demographic and Health Survey program, which provides standardized data on population health, household conditions, and socioeconomic characteristics across Nepal. The survey covered all seven provinces, including urban and rural areas. Standardized questionnaires were used by trained field staff, and data collection followed strict ethical considerations and quality control [30].

### 2.2 Sampling and Sample Size

**Figure 1:**
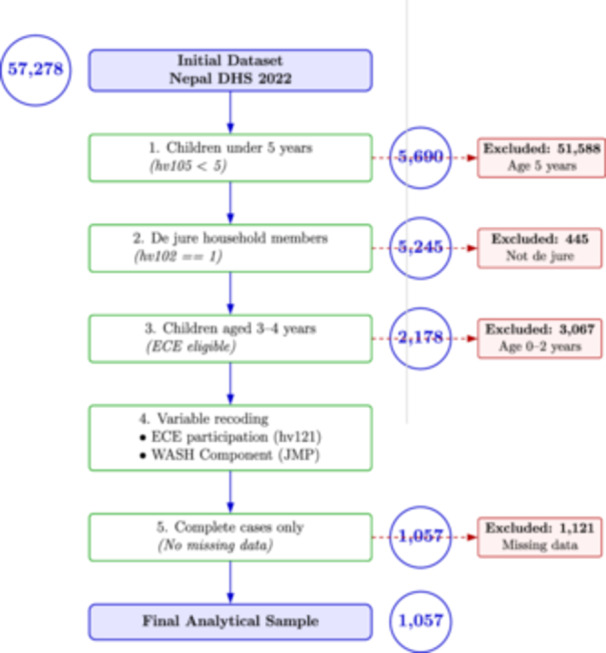
Flowchart of study sample selection and analytical steps for WASH and ECE participation in Nepal.

The analysis included de jure household members aged 3–4 years, representing children eligible for ECE programs. The NDHS used a stratified two‐stage cluster sampling design, with primary sampling units selected proportional to size, followed by systematic household selection within clusters. Children below 3 years were excluded to avoid misclassification, as most would not have joined formal early learning programs. From the initial DHS dataset (*N* = 57,278), 2178 children met the age eligibility criterion. After applying the de jure residency criterion and excluding observations with missing outcome or primary exposure variables, the final analytical sample comprised 1057 children. Figure 1 illustrates the flow from the age‐eligible sample to the final analytical sample, highlighting the impact of de jure versus de facto residency and missing data exclusions. Descriptive comparisons of basic demographics between included and excluded children indicated no substantial differences, suggesting the analytical sample is broadly representative of the eligible population (Figure 1).

### 2.3 Outcome Variable

The outcome of interest was ECE participation, defined as attending any organized ECE as a pre‐primary program during the current school year, and derived from the NDHS variable hv121 coded as a binary variable: 1 = Yes, 0 = No. Although in standard DHS datasets the hv121 variable is often skipped for children younger than 5 years, in the NDHS dataset this variable was available for children aged 3–4 years and was therefore used as the closest proxy indicator of participation in organized early childhood education programs. This approach is consistent with the DHS methodological guidelines, where the school attendance variable (hv121) is commonly used to identify participation in organized learning or pre‐primary education when educational level information is considered [34]. Therefore, hv121 was used to represent ECE attendance in the present analysis.

### 2.4 Exposure Variables

The primary exposure of interest in this study was the household handwashing environment, conceptualized as a binary composite variable as per WHO/UNICEF JMP (Joint Monitoring Program) definitions. Households were coded as adequate (1) if water was available at a fixed handwashing facility inside the dwelling or yard and as limited (0) if water was absent or the facility was mobile. The classification of WASH indicators followed the standard definitions of the WHO/UNICEF JMP to ensure international comparability. Other WASH component variables of interest included drinking water source (improved vs. unimproved) and water treatment (treated vs. untreated) [35].

### 2.5 Covariates

The covariates were selected from the previous literature and theoretical relevance: child characteristics‐ sex, birth order; maternal characteristics‐ education level: no education, primary, and higher household characteristics‐ household size, sex of household head, wealth status, access to mass media; and contextual factors: residence, urban/rural, administrative province [32, 36, 37, 38, 39, 40].

### 2.6 Cleaning and Handling Missing Data

This study restricted the dataset to de jure household members and recoded variables into binary or categorical formats following NDHS guidelines. Using a complete‐case approach, observations with missing outcome or primary exposure variables were excluded, and sensitivity analyses were conducted to evaluate potential impacts of missing data [41]. The remainder of the covariates was explored for missingness. Approximately 50% of eligible children were excluded due to missing data. To assess potential selection bias, we compared basic demographic characteristics (age, sex, residence, and household wealth) between included and excluded children and found no substantial differences, suggesting that the analytical sample is broadly representative of the eligible population [42]. This approach is consistent with standard guidelines for handling missing survey data [43]. Nevertheless, the exclusion of children with missing information remains a limitation, and results should be interpreted with this consideration in mind.

### 2.7 Bias and Confounding

All the analyses involved the use of a nationally representative sample and restriction to de jure household members, which minimizes selection bias. Measurement bias was low because standardized NDHS questionnaires were utilized, with JMP definitions of WASH indicators. We have controlled for potential confounding by including conceptually relevant covariates in our multivariable logistic regression models. Because the data are cross‐sectional, the analysis focuses on statistical associations rather than causal inference.

### 2.8 Confounder Selection and Model Development Strategies

This study used a conceptually directed and data‐driven method. First, covariates were selected based on theoretical significance, previous studies, and accessibility in the dataset. Second, bivariate analyses using chi‐square tests recognized variables associated with ECE participation (*p* < 0.20) for potential inclusion. Third, univariate analyses to examine the odds ratio change in estimators (CIE) and assumed potential confounders. Finally, multivariable logistic regression models included both conceptually significant and statistically related variables to identify independent risk factors. All statistical tests were two‐sided, and statistical significance was assessed at a predefined alpha level of 0.05.

### 2.9 Sampling Weights and Survey Design

All analyses complex‐survey‐designed for stratification, clustering, and sampling weights. Cluster‐adjusted weights were applied to ensure nationally representative estimates and correct for unequal probabilities of selection. Survey design variables, including sampling weights, clustering, and stratification, were incorporated using survey‐adjusted estimation procedures to obtain unbiased population estimates.

### 2.10 Analytical Framework

#### 2.10.1 Multivariable Logistic Regression Model

Let $Y_{i}$denote the binary outcome for child i, where:

$$Y_{i}=\left\{\begin{array}{ll} 1, & \text{if child attended any ECE program}\\ 0, & \text{otherwise} \end{array}\right.$$

The survey‐weighted multivariable logistic regression model is:

$$\text{logit}(P(Y_{i}=1))=\beta_{0}+\sum_{k=1}^{K}\beta_{k}X_{\mathrm{ik}}$$

Where $X_{\mathrm{ik}}$represents the following predictors:

Household WASH components, contextual, geographical, maternal, child, media access, and other covariates were derived from the statistical and conceptual framework. Here, $\beta_{0}$is the intercept, and $\beta_{k}$are the regression coefficients corresponding to each predictor. Exponentiating $\beta_{k}$provides adjusted odds ratios (AORs) for ECE participation, controlling for all other covariates.

### 2.11 Survey Weighted Logistic Regression by Residence Subgroup

#### 2.11.1 Rural Households

For children in rural households (residence=0), the probability of ECE participation ($Y_{i}=1$) was modeled as:

$$\text{logit}(P(Y_{i}=1|\text{residence}=0))=\beta_{0}^{\left(R\right)}+\beta_{1}^{\left(R\right)}i\text{.}\text{water\_imp}+\beta_{2}^{\left(R\right)}i.\text{water\_treat}+\beta_{3}^{\left(R\right)}i.\text{handwash\_env}+\beta_{4}^{\left(R\right)}i.\text{division}+\beta_{5}^{\left(R\right)}\text{hh\_size}+\beta_{6}^{\left(R\right)}i.\text{mother\_edu}+\beta_{7}^{\left(R\right)}i.\text{birth\_order\_cat}+\beta_{8}^{\left(R\right)}i.\text{media\_access}+\beta_{9}^{\left(R\right)}i.\text{wealth}+\beta_{10}^{\left(R\right)}i.\text{child\_sex}$$

Here:

- $\beta_{0}^{\left(R\right)}$is the intercept for rural households

- $\beta_{1}^{\left(R\right)}$–$\beta_{10}^{\left(R\right)}$ are regression coefficients for each predictor

- All categorical variables included reference categories (Ref).

#### 2.11.2 Urban Households

For children in urban households (residence=1), the model is similarly specified:

$$\text{logit}(P(Y_{i}=1|\text{residence}=1))=\beta_{0}^{\left(U\right)}+\beta_{1}^{\left(U\right)}i.\text{water\_imp}+\beta_{2}^{\left(U\right)}i.\text{water\_treat}+\beta_{3}^{\left(U\right)}i.\text{handwash\_env}+\beta_{4}^{\left(U\right)}i.\text{division}+\beta_{5}^{\left(U\right)}\text{hh\_size}+\beta_{6}^{\left(U\right)}i.\text{mother\_edu}+\beta_{7}^{\left(U\right)}i.\text{birth\_order\_cat}+\beta_{8}^{\left(U\right)}i.\text{media\_access}+\beta_{9}^{\left(U\right)}i.\text{wealth}+\beta_{10}^{\left(U\right)}i.\text{child\_sex}$$

Here:

- $\beta_{0}^{\left(U\right)}$is the intercept for urban households.
- $\beta_{1}^{\left(U\right)}$–$\beta_{10}^{\left(U\right)}$ are regression coefficients for urban children.
- Categorical predictors use reference categories as a baseline.

Where:

These subgroup models allow comparison of WASH and socio‐demographic predictors of ECE participation within rural versus urban contexts.

- Exponentiating $\beta_{k}^{\left(R\right)}$or $\beta_{k}^{\left(U\right)}$gives adjusted odds ratios (AORs) with 95% confidence intervals.
- Survey weights, clustering, and stratification were applied in both subgroup analyses.

### 2.12 Statistical Analysis

This study used descriptive statistics and several bar charts to summarize WASH components, household, maternal, and child characteristics by ECE participation. It evaluated the initial association between all categorical variables using chi‐square tests. Survey‐weighted logistic regression models estimated crude and adjusted odds ratios (OR) with 95% confidence intervals (CIs). Variables which proved significant in bivariate analysis (*p* < 0.05) or of conceptual importance were included in multivariable models to identify independent predictors of ECE participation. All analyses were performed using survey‐adjusted estimation procedures. Besides, a separate multivariable logistic regression model for rural‐urban stratified analysis. Additionally, a spatial choropleth map to assess the province‐wise spatial variation of ECE. All analyses were performed using STATA version 17 and RStudio version 4.5.1. Statistical reporting adhered to the STROBE (Strengthening the Reporting of Observational Studies in Epidemiology) guidelines for observational health research [44]. Hypothesis testing, including P values, was reported with effect sizes and confidence intervals following standard conventions (two‐sided tests, *α* = 0.05; *p* < 0.001, P 0.001–0.01 to the nearest thousandth, *p* ≥ 0.01 to the nearest hundredth). All statistical terms and abbreviations are defined at first use, pre‐specified analyses are distinguished from exploratory/subgroup analyses, and reporting followed SAMPL guidelines [45].

### 2.13 Model Assumptions and Diagnosis

This study employed the discriminatory ability of the logistic regression models was evaluated using the area under the receiver operating characteristic curve (AUC–ROC). Besides, the Hosmer–Lemeshow (H–L) test is a statistical test used to evaluate the goodness‐of‐fit for logistic regression models. Additionally, the Variance Inflation Factor (VIF) was assumed to be less than 10 as there was no multicollinearity among all independent variables.

### 2.14 Adjusted Covariates on Final Models

**Figure 2:**
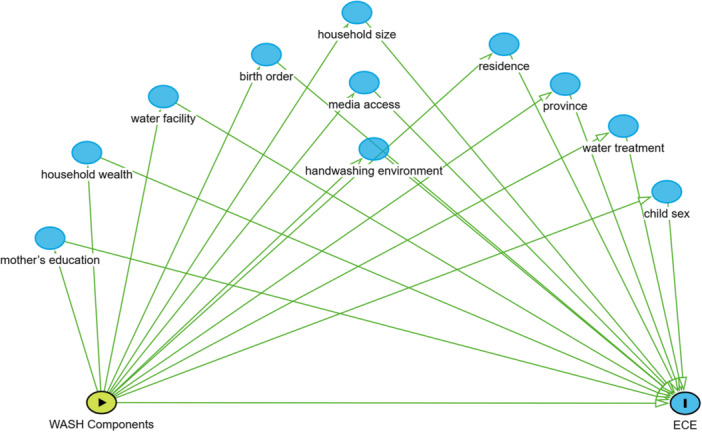
Conceptual DAG of handwashing environment and ECE enrollment.

This set of covariates was included in models such as water facility, water treatment, handwashing environment, province, household size, birth order, media access, residence, household wealth, and child sex (Figure 2).

### 2.15 Ethical Considerations

The data used in this study were derived from a nationally representative household survey conducted in Nepal that received ethical approval from the Nepal Health Research Council (NHRC) and the Institutional Review Board (IRB) of the survey‐implementing organization. Written informed consent was obtained from all adult participants and from parents or legal guardians on behalf of participating children at the time of data collection. Details at DHS are available at:https://dhsprogram.com/Methodology/Protecting-the-Privacy-of-DHS-Survey-Respondents.cfm.

## 3 Results

### 3.1 Descriptive Association Between Household WASH Conditions and ECE

**Figure 3:**
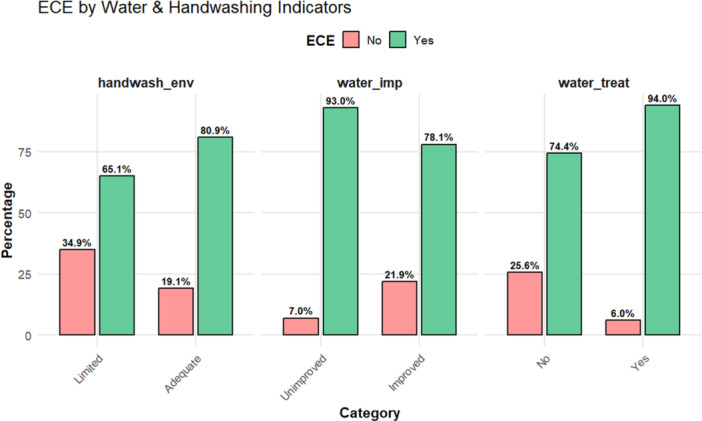
ECE Participation by Household WASH Indicators.

Children from households with access to better water and hand‐washing facilities showed much higher chances of enrolling in early childhood education compared to those from households with limited access to such facilities There was an increased enrollment in early childhood education among households with proper hand‐washing facilities (81% and 65%), access to an improved water source (78% and 7%), and treated water in the household (94% and 74%) compared to those who did not have access to such facilities. These descriptive patterns suggest that children living in households with improved WASH conditions tend to show higher participation in early childhood education programs (Figure 3).

### 3.2 Background Characteristics and Distribution of ECE Enrollment

Of the 1057 children, 832 (or 78.7%) were enrolled in early childhood education, while 225 (or 21.3%) were not enrolled in ECE. This indicates that a substantial proportion of children in the analytical sample were already participating in organized early childhood education programs.

### 3.3 Household WASH Conditions and ECE Participation

ECE enrollment was significantly higher when there was improved access to water sources (74.9% vs. 3.8% for unimproved; *p* = 0.019) and treated household drinking water (20.7% vs. 58.0% for untreated; *p* < 0.001). Additionally, children were more likely to attend ECE when their households had basic facilities for handwashing (70.7% vs. 8.0% for limited; *p* = 0.003) and a fixed facility for handwashing (73.0% vs. 5.7% for mobile; *p* < 0.001). Overall, when there were adequate handwashing facilities, there was a strong association with enrollment. These findings highlight the potential role of household hygiene environments in supporting children's participation in early learning programs.

### 3.4 Socio‐Demographic Determinants of ECE

Provincial inequalities were observed, with Karnali (14.0%) and Koshi (13.9%) having the highest enrollment and Gandaki with the lowest (7.1%) (*p* < 0.001). Households with more than 5 members had more enrollment (48.3%) than those with fewer than 5 household members (30.4%) (*p* < 0.001). Birth order was an important variable, with first children having lower enrollment (12.8%) than later‐born children (*p* = 0.047). Mass media showed higher enrollment (76.6%) than those with no mass media (Table 1). These results indicate that household socioeconomic and informational environments may play an important role in shaping ECE participation.

**Table 1:** Characteristics of children by ECE enrollment and WASH status.

Characteristics	ECE enrollment		
	No	Yes	*p* value
	*N* (%)	*N* (%)	
	225 (21.29)	832 (78.71)	
Source of water			
Unimproved	3 (0.28)	40 (3.78)	0.019
Improved	222 (21.00)	792 (74.93)	
Treatment of household drinking water			
No	211 (19.96)	613 (57.99)	< 0.001
Yes	14 (1.32)	219 (20.72)	
Handwashing basics facility			
Limited	39 (3.69)	85 (8.04)	0.003
Basic	186 (17.60)	747 (70.67)	
Handwashing place			
Fixed	186 (17.60)	772 (73.04)	< 0.001
Mobile	39 (3.69)	60 (5.68)	
Handwash Environment			
Limited/no	51 (4.82)	95 (8.99)	< 0.001
Adequate/yes	174 (16.46)	737 (69.73)	
Residence			
Rural	111 (10.50)	438 (41.44)	0.378
Urban	114 (10.79)	394 (37.28)	
Division			
Koshi	23 (2.18)	147 (13.91)	< 0.001
Madhesh	100 (9.46)	128 (12.11)	
Bagmati	16 (1.51)	109 (10.31)	
Gandaki	6 (0.57)	75 (7.10)	
Lumbini	21 (1.99)	116 (10.97)	
Karnali	36 (3.41)	148 (14.00)	
Sudurpashchim	23 (2.18)	109 (10.31)	
Wealth Index			
Poorest	98 (9.27)	297 (28.10)	0.112
Poorer	46 (4.35)	157 (14.85)	
Middle	41 (3.88)	171 (16.18)	
Richer	23 (2.18)	123 (11.64)	
Richest	17 (1.61)	84 (7.95)	
Household Size			
< 5 members	55 (5.20)	321 (30.37)	< 0.001
≥ 5 members	170 (16.08)	511 (48.34)	
Household head's sex			
Female	75 (7.10)	304 (28.76)	0.374
Male	150 (14.19)	528 (49.95)	
Birth Order Category			
1	23 (2.18)	135 (12.77)	0.047
2–3	50 (4.73)	149 (14.10)	
4+	152 (14.38)	548 (51.84)	
Child Sex			0.067
Female	97 (9.18)	416 (39.36)	
Male	128 (12.11)	416 (39.36)	
Access to mass media			
No access	18 (1.70)	22 (2.08)	< 0.001
Has access	207 (19.58)	810 (76.63)	

### 3.5 Logistic Regression Analysis of Factors Associated With ECE Enrollment

In survey‐weighted logistic regressions, some variables were found to be significantly associated with ECE enrollment. In unadjusted logistic regressions, ECE participation was found significantly associated with improved water source (AOR = 0.16, 95% CI: 0.04–0.60; *p* = 0.006), treated drinking water (AOR = 6.37, 95% CI: 3.39–11.96; *p* < 0.001), adequate hand washing environment (AOR = 2.33, 95% CI: 1.47–3.70; *p* < 0.001), fixed hand washing place (OR 2.75, 95% CI: 1.73–4.38; *p* < 0.001), basic hand washing facilities (AOR = 1.70, 95% CI: 1.02–2.83; *p* = 0.041), wealth (richer: AOR = 2.36, 95% CI: 1.33–4.19; *p* = 0.004).

However, when adjusting for possible covariates, only a group of variables remained statistically significant. Media availability also had a positive association (AOR = 5.17, 95% CI: 2.04–13.10; *p* = 0.001), while those living in rural areas had reduced odds of enrollment (AOR = 0.51, 95% CI: 0.28–0.92; *p* = 0.027). In the wealth group, children from richer communities had increased odds of ECE attendance compared with those from the poorest (AOR = 2.61, 95% CI: 1.04–6.52; *p* = 0.040) (Table 2). After controlling for socioeconomic and contextual covariates, the influence of several WASH variables was attenuated, suggesting that household socioeconomic conditions may partly explain their relationship with ECE participation.

**Table 2:** Household WASH and socio‐demographic factors associated with ECE enrollment (*N* = 1057).

Characteristics	Survey‐weighted logistic Regression			
	Crude OR (95% CI)	*p* value	Adjusted OR (95% CI)	*p* value
Source of water				
Improved	0.16 (0.04–0.60)	0.006	0.26 (0.05–1.47)	0.127
Unimproved (Ref)				
Treatment of household drinking water				
Yes	6.37 (3.39–11.96)	< 0.001	1.52 (0.62–3.72)	0.360
No (Ref)				
Handwashing environment				
Adequate	2.33 (1.47–3.70)	< 0.001	2.08 (0.31–14.05)	0.450
Inadequate (Ref)				
Handwashing place				
Fixed	2.75 (1.73–4.38)	< 0.001	0.63 (0.11–3.49)	0.598
Not fixed (Ref)				
Handwashing basic				
Yes	1.70 (1.02–2.83)	0.041	1.26 (0.33–4.86)	0.737
No				
Division				
Madhesh	0.22 (0.12–0.43)	< 0.001	0.29 (0.12–0.71)	0.007
Bagmati	1.69 (0.72–3.95)	0.224	1.06 (0.33–3.43)	0.925
Gandaki	2.27 (0.73–7.04)	0.155	1.84 (0.31–10.80)	0.498
Lumbini	0.90 (0.43–1.91)	0.786	0.96 (0.32–2.87)	0.947
Karnali	0.82 (0.41–1.64)	0.576	1.74 (0.67–4.49)	0.255
Sudurpashchim	0.76 (0.34–1.70)	0.500	0.62 (0.19–2.06)	0.437
Koshi (Ref)				
Residence				
Rural	0.83 (0.56–1.21)	0.331	0.51 (0.28–0.92)	0.027
Urban (Ref)				
Wealth				
Poorer	1.23 (0.73–2.05)	0.439	1.61 (0.75–3.43)	0.221
Middle	1.50 (0.96–2.34)	0.076	2.10 (0.95–4.63)	0.066
Richer	2.36 (1.33–4.19)	0.004	2.61 (1.04–6.52)	0.040
Richest	2.10 (1.04–4.22)	0.037	1.64 (0.41–6.56)	0.486
Poorest (Ref)				
Household size				
≥ 5	0.44 (0.30–0.65)	< 0.001	0.66 (0.35–1.24)	0.197
< 5 members (Ref)				
No education				
Birth order				
2–3	0.64 (0.35–1.17)	0.145	0.55 (0.21–1.45)	0.230
4+	0.70 (0.43–1.15)	0.161	0.71 (0.34–1.47)	0.358
Birth order 1 (Ref)				
Child sex				
Male	0.83 (0.60–1.14)	0.253	0.69 (0.40–1.18)	0.175
Female				
Media access				
Has access	3.89 (1.84–8.25)	< 0.001	5.17 (2.04–13.10)	0.001
No access (Ref)				

*Note:* Odds ratios (ORs) with 95% confidence intervals (CIs) are presented. All models were adjusted for water facility, water treatment, handwashing environment, province, household size, mother's education, birth order, media access, residence, household wealth, and child sex. Reference categories are indicated as “Ref”. Survey weights, strata, and primary sampling units were applied.

### 3.6 Model Performance and Predictive Accuracy

**Figure 4:**
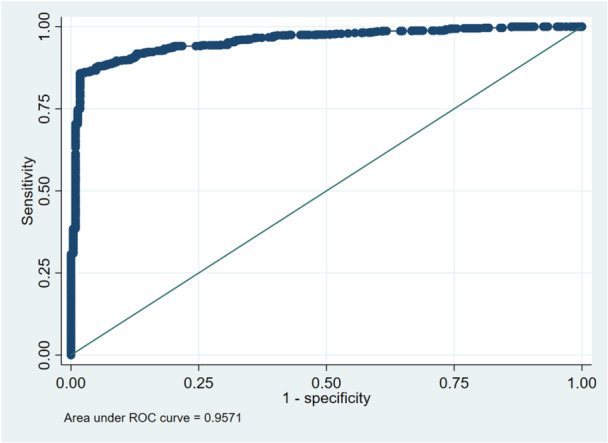
Predicting ECE participation.

From the ROC curve, it can be observed that the discrimination power of the predicting model regarding children who attend ECE and those who do not attend is very good. This is because the ROC curve is well above the diagonal reference line. Another confirmation of the excellent discriminatory power of the predicting model can be derived from the AUC value. This value is 0.957. This means that the predicting model will correctly choose a higher predicted probability for children who attend ECE instead of children who do not attend ECE about 96% of the time (Figure 4). This indicates excellent model discrimination and suggests that the selected predictors collectively explain a substantial proportion of variation in ECE participation.

### 3.7 Stratified Analysis by Place of Residence

Stratified analysis by residence revealed that mother's education and media exposure were independent predictor of early childhood education enrollment in both rural and urban areas. Media use was positively associated with enrollment in rural (AOR = 3.84, 95% CI: 1.27–11.60; *p*= 0.017) and urban (AOR = 6.25, 95% CI: 1.75–22.28; *p*= 0.005) areas.

The wealth index showed a differential effect with residence. That is, within the urban areas, children from the middle and richer wealth index showed higher odds of being enrolled in school than those from the poorest households (middle AOR = 6.43, CI: 1.42–29.2; richer AOR = 8.73, CI: 2.01–37.95; *p* < 0.05). This pattern suggests that economic inequality may play a stronger role in shaping ECE participation in urban settings compared with rural areas. Overall, the findings indicate that media exposure and household wealth are important determinants of ECE participation, irrespective of residence, while wealth appears to exert a stronger influence in urban environments. These results imply that socioeconomic and informational factors may have a stronger influence on ECE participation than household WASH variables once other determinants are considered (Table 3).

**Table 3:** Adjusted odds ratios for factors associated with ECE Enrollment by place of residence (urban *N* = 508, rural *N* = 548).

Characteristics	Survey weighted multivariable logistic regression			
	Rural AOR (95% CI)	*p* value	Urban AOR (95% CI)	*p* value
Water improved				
Improved	0.24 (0.03–1.99)	0.187	0.29 (0.03–3.25)	0.311
Unimproved (Ref)				
Water treatment				
Yes	1.66 (0.61–4.48)	0.315	1.09 (0.24–5.06)	0.909
No (Ref)				
Handwashing environment				
Adequate	2.29 (0.79–6.63)	0.125	1.22 (0.31–4.82)	0.776
Inadequate (Ref)				
Division				
Madhesh	0.31 (0.06–1.44)	0.133	0.31 (0.08–1.14)	0.078
Bagmati	0.57 (0.13–2.51)	0.458	1.20 (0.15–9.68)	0.862
Gandaki	1.44 (0.25–8.37)	0.685	2.33 (0.09–57.34)	0.604
Lumbini	0.51 (0.11–2.35)	0.383	0.97 (0.19–4.92)	0.975
Karnali	1.00 (0.22–4.62)	0.997	3.82 (0.72–20.14)	0.113
Sudurpashchim	0.28 (0.05–1.57)	0.146	0.84 (0.15–4.71)	0.844
Koshi (Ref)				
Household size				
≥ 5 members	0.64 (0.30–1.36)	0.243	0.71 (0.25–1.99)	0.515
< 5 members (Ref)				
Birth order				
2–3	0.42 (0.11–1.64)	0.211	0.76 (0.19–3.04)	0.695
Birth order 4 +	0.56 (0.18–1.80)	0.332	0.92 (0.31–2.75)	0.884
Birth order 1 (Ref)				
Media access				
Has access	3.84 (1.27–11.60)	0.017	6.25 (1.75–22.28)	0.005
No access (Ref)				
Wealth index				
Poorer	0.41 (0.12–1.45)	0.167	6.55 (1.57–27.42)	0.010
Middle	1.32 (0.39–4.43)	0.649	6.43 (1.42–29.20)	0.016
Richer	1.18 (0.28–4.99)	0.817	8.73 (2.01–37.95)	0.004
Richest	0.11 (0.02–0.49)	0.004	6.02 (0.80–45.34)	0.081
Poorest (Ref)				
Child sex				
Male	0.98 (0.45–2.14)	0.962	0.52 (0.24–1.12)	0.094
Female (Ref)				

*Note:* Odds ratios (ORs) with 95% confidence intervals (CIs) are presented. All models were adjusted for water facility, water treatment, handwashing environment, province, household size, birth order, media access, residence, household wealth, and child sex. Reference categories are indicated as “Ref”. Survey weights, strata, and primary sampling units were applied.

### 3.8 Spatial Distribution of ECE Enrollment Across Provinces

**Figure 5:**
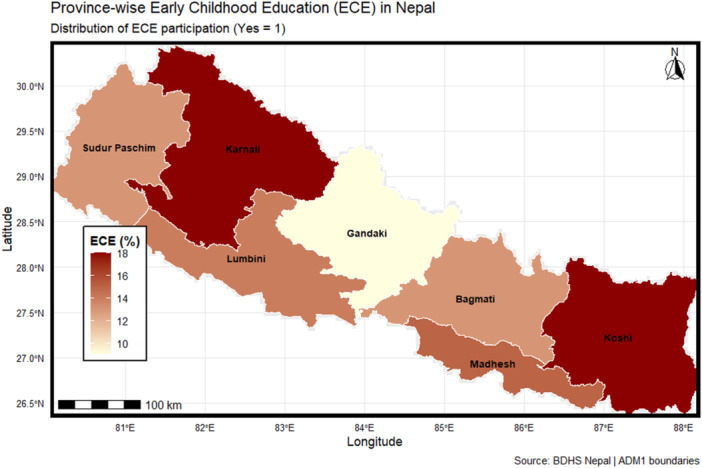
Province‐wise spatial distribution of Early Childhood Education (ECE) participation in Nepal.

On the spatial map, provinces show varying levels of early childhood education (ECE) enrollment across Nepal. Karnali and Koshi provinces showed relatively higher levels of ECE participation, whereas Gandaki province demonstrated comparatively lower enrollment levels. These spatial variations highlight potential geographic inequalities in access to early childhood education services across provinces in Nepal (Figure 5).

## 4 Discussion

This study on Nepali households found that children from households with better water supply points, arsenic‐reduced drinking water, and improved hand‐washing facilities were more likely to have higher unadjusted rates of participation in early childhood education (ECE) programs. These findings suggest that the observed relationship between WASH facilities and ECE participation may partly reflect broader household socioeconomic advantages rather than a direct causal pathway. Later analysis with the adjustment of socio‐demographic variables again identified the significant predictors for participation in ECE to be household media exposure. Results also revealed the presence of significance for ECE participation for children living in richer households and living in rural areas. Large regional disparities were identified by provinces; the regions of Karnali and Koshi have the highest levels of participation for ECE programs, and lower participation levels for the Gandaki province.

The above‐unadjusted relationships between WASH infrastructure and enrollment in ECE might be influenced by wider societal and economic factors [46]. Moreover, exposure to mass media might yield information that could inform parents about their enrollment in ECE programs [47]. Mass media may also influence parental perceptions regarding the benefits of early education and increase awareness of available educational services, particularly in resource‐limited settings [19]. Moreover, the differential impact of wealth in urban versus rural areas has confirmed that although infrastructure has some significance, social and economic assets and parental understanding remain fundamental to ECE enrollment [48]. One possible explanation is that early childhood education services in rural areas may be more uniformly distributed through community‐based or public education initiatives, resulting in relatively similar participation across wealth groups [49]. In contrast, urban areas often present greater socioeconomic stratification, where access to higher‐quality or private ECE facilities may depend more strongly on household financial capacity [50]. After adjusting for socio‐demographic covariates, the attenuation of WASH effects indicates that social determinants such as media access and household wealth may play a stronger role in shaping early educational participation [51]. The high predictive accuracy of the model (AUC = 0.957) further supports the robustness of the selected socio‐demographic predictors in explaining ECE participation patterns [52]. This exceptionally high AUC may partly reflect the strong predictive influence of socio‐economic indicators such as media access and household wealth, which can act as proxies for parental awareness and access to educational opportunities [53, 54]. However, since the model incorporates multiple socio‐demographic covariates derived from a nationally representative dataset, the likelihood that the high predictive performance is solely due to overfitting is reduced [55]. Provincial disparities highlight that policy implementation and regional infrastructure strongly influence ECE participation [56].

The findings are supported by previous literature from South Asia and developing countries, which had identified media access and household wealth as fundamental parameters influencing ECE enrollment [23, 25, 47]. This study offers a contribution to this literature by showing that WASH infrastructure is indeed associated with ECE enrollment, but such associations seem to be explained by both socio‐economic statuses [22, 57]. This suggests that improved WASH facilities may function as a proxy indicator of overall household living conditions and parental capacity to support early educational enrollment [58, 59, 60, 61]. This is in support of the hypothesis suggesting potential explanatory roles for health and hygiene policies in influencing educational enrollment rather than serving as direct influencers [60, 62, 63]. The urban‐rural gaps in both WASH infrastructure and ECE enrollment appear consistent with previous literature suggesting enhanced infrastructure would benefit urban children from more affluent backgrounds, while rural children would remain at a disadvantaged position despite enhanced infrastructure [56, 64, 65, 66, 67, 68].

## 5 Conclusion

The role of household access to media in stimulating the enrollment of early childhood education (ECE) is highly important in Nepal, and the results are consistent between rural and urban environments. Although household water, sanitation, and hygiene (WASH) components infrastructure is critical to child health, it has a seemingly significant effect on ECE participation, but is mostly explained by parental and socio‐economic influences. The inequalities in wealth are mostly experienced by the urban children, and in rural regions, there are more structural and informational barriers that require contextual interventions. The provincial differences depict a high level of spatial inequity, where Karnali and Koshi have a higher level of enrollment service demands in comparison to Gandaki, which has a low level of participation, meaning that resource allocation and target outreach should be based on regional disparities. Findings are in line with Sustainable Development Goal 4 (inclusive and equitable quality education) and add to the progression of Millennium Development Goal 2 (universal primary education) via education equity and access at an early age. Policy measures must focus on media‐based awareness and integrated interventions, involving health, hygiene, and educational assistance. EEC can be used to boost the rural disadvantages and provincial inequalities by developing tailored programs that would boost participation in the ECE, the developmental outcomes, and strengthen the foundation towards a lifetime of learning. Our results can be used to inform policy responses by connecting social determinants, spatial conditions, and educational policy to inform equitable early childhood development programs in Nepal and other low‐ and middle‐income countries.

## 6 Limitations

There are a few limitations to be considered in this study. These findings remain non‐causal, as the research design is cross‐sectional. Furthermore, uncontrolled variables may exist despite multiple regressions. It is also possible that the WASH facilities being assessed at the household level do not provide complete information about functionality, use rates, behavioral acceptances, or the biological quality of water. Additionally, the survey captures only the presence of WASH facilities at a single point in time, rather than their consistent functionality or actual usage. Additionally, the cross‐sectional design prevents establishing temporal relationships between WASH access and ECE enrollment. Maternal education and age were not included in the main analysis because over 50% of the data for these variables were missing. Moreover, within these provinces, variability in findings might be masked. There is also a possibility of self‐reported bias, as ECE and household factors are assessed.

## 7 Policy Recommendations

These results have important policy implications for interventions designed to encourage enrollment in ECE. Programs that can boost maternal education and communicate focused information through mass media may have great potential, especially in rural and poorer areas. Community‐based awareness campaigns and digital media outreach may therefore be effective strategies for improving parental knowledge regarding the benefits of early childhood education. In addition, since household wealth appears to be a stronger barrier to ECE participation in urban areas, targeted policy measures such as urban ECE subsidies or financial support programs may help reduce economic inequalities in access. Strengthening regulatory frameworks and promoting affordable, low‐cost private ECE providers in cities may also improve equitable access for children from lower‐income households. Although continued investment in WASH infrastructure is necessary for childhood health and development in general, combined interventions that can address both factors of media engagement and infrastructure development may help create more favorable conditions for ECE enrollment. Further research is needed to explore potential pathways and the usefulness of combined interventions that can focus on both health and education for improved ECE enrollment.

## 8 Theoretical Contributions

The current study makes an addition to theoretical models of the role of social factors in early childhood education by providing an understanding of the mediator function of parents’ educational attainment and socio‐economic status with regard to the connection between the environmental infrastructure and enrollment in ECE programs. The use of differential effects of wealth across urban and rural areas also brings into focus the significance of context‐driven factors in defining accessibility and enrollment in educational institutions.

## Author Contributions

**Md Salek Miah:** conceptualization, investigation, writing – original draft, writing – review and editing, visualization, validation, methodology, software, formal analysis, project administration, data curation, supervision, resources. **Rima Akhter:** supervision, writing – review and editing, validation, project administration. **Fhameda Faija Lamia, Mst. Labonna Begum, Md Shied, and Rima Akhter:** equally contributed to write and literature review.

## Funding

The authors have nothing to report.

## Consent

The authors have nothing to report.

## Conflicts of Interest

The authors declare no conflicts of interest.

## Declaration of Generative AI and AI‐Assisted Technologies in the Writing Process

The authors utilized generative AI tools like Grammarly, QuillBot, and ChatGPT (GPT‐4) just to spotlessly correct the language and make the writing flatter. They didn't use AI for any data work, no generating, analyzing, or interpreting.

## Transparency Statement

The lead author, Md Salek Miah, affirms that this manuscript is an honest, accurate, and transparent account of the study being reported; that no important aspects of the study have been omitted; and that any discrepancies from the study as planned (and, if relevant, registered) have been explained.

## Data Availability

This secondary data is publicly available from the DHS program website upon request (https://www.dhsprogram.com/data) for researchers who meet the criteria for access. The code used to perform all analyses in this study is publicly available on GitHub https://github.com/muhammadsalek/Nepal_ECE_Analysis/blob/main/README.md.
